# Missing data approaches in longitudinal studies of aging: A case example using the National Health and Aging Trends Study

**DOI:** 10.1371/journal.pone.0286984

**Published:** 2023-06-08

**Authors:** Emilie D. Duchesneau, Shahar Shmuel, Keturah R. Faurot, Allison Musty, Jihye Park, Til Stürmer, Alan C. Kinlaw, Yang Claire Yang, Jennifer L. Lund

**Affiliations:** 1 Department of Epidemiology, Gillings School of Global Public Health, University of North Carolina at Chapel Hill, Chapel Hill, North Carolina, United States of America; 2 Department of Physical Medicine and Rehabilitation, School of Medicine, University of North Carolina, Chapel Hill, North Carolina, United States of America; 3 Division of Pharmaceutical Outcomes and Policy, University of North Carolina School of Pharmacy, Chapel Hill, North Carolina, United States of America; 4 Cecil G. Sheps Center for Health Services Research, University of North Carolina at Chapel Hill, Chapel Hill, North Carolina, United States of America; 5 Department of Sociology, Carolina Population Center, Lineberger Cancer Center, University of North Carolina at Chapel Hill, Chapel Hill, North Carolina, United States of America; National Institute of Environmental Health Sciences, UNITED STATES

## Abstract

**Purpose:**

Missing data is a key methodological consideration in longitudinal studies of aging. We described missing data challenges and potential methodological solutions using a case example describing five-year frailty state transitions in a cohort of older adults.

**Methods:**

We used longitudinal data from the National Health and Aging Trends Study, a nationally-representative cohort of Medicare beneficiaries. We assessed the five components of the Fried frailty phenotype and classified frailty based on their number of components (robust: 0, prefrail: 1–2, frail: 3–5). One-, two-, and five-year frailty state transitions were defined as movements between frailty states or death. Missing frailty components were imputed using hot deck imputation. Inverse probability weights were used to account for potentially informative loss-to-follow-up. We conducted scenario analyses to test a range of assumptions related to missing data.

**Results:**

Missing data were common for frailty components measured using physical assessments (walking speed, grip strength). At five years, 36% of individuals were lost-to-follow-up, differentially with respect to baseline frailty status. Assumptions for missing data mechanisms impacted inference regarding individuals improving or worsening in frailty.

**Conclusions:**

Missing data and loss-to-follow-up are common in longitudinal studies of aging. Robust epidemiologic methods can improve the rigor and interpretability of aging-related research.

## Introduction

The United States is experiencing unprecedented growth in its aging population, largely due to the aging of the Baby Boomer generation and advancements in sanitation and medicine [1]. Population aging presents numerous public health challenges, as older adults face elevated risks of health complications and have higher healthcare utilization and spending. High-quality longitudinal research is necessary for identifying interventions that can promote healthy aging and well-being during the later years of life.

Longitudinal studies of aging are prone to methodological challenges. Higher attrition for older adults (by death or loss-to-follow-up) can induce selection bias since this attrition is often informative. Additionally, studies of older adults may be prone to missing data bias. Data on important geriatric syndromes may be missing not at random (MNAR), which means that the missing data mechanism depends on unobserved values (e.g., cognitively-impaired individuals may be less likely to participate in cognitive assessments than their counterparts) [2, 3]. Missing data bias and selection bias are not limited to studies of aging, but these issues are particularly relevant in studies of older adults where follow-up and data collection depend on unique healthcare issues such as comorbidities, cognitive impairment, and/or frailty.

One setting in which these methodological challenges may occur is in studies describing frailty state transitions. Frailty is a dynamic age-related state characterized by reduced physiological homeostasis and vulnerability to physiological decline, disability, adverse health outcomes, and death [4–7]. Frailty is dynamic [8]; a recent international meta-analysis found that over an average follow-up of 3.9 years for older adults, approximately 10% experienced improvements in frailty, 40% experienced worsening, and 50% experienced no change [9]. However, many of the component studies of the systematic review implemented a complete case analysis or otherwise did not appropriately account for missing data. If individuals with missing data have different frailty trajectories than those without missing data, conducting a complete case analysis is expected to bias findings from these studies. For example, if individuals who are lost-to-follow-up are inherently different (e.g., more frail) than those who remain under observation, studies using a complete case analysis may result in fewer transitions to prefrail or frail states over time.

In this paper, we describe potential solutions to account for selection bias due to differential attrition and missing data bias in studies of aging. We apply these methods to a study describing one-, two-, and five-year frailty state transitions in a large and diverse cohort of older adults (≥65 years) in the United States.

## Materials and methods

We present a descriptive study of frailty state transitions using the National Health and Aging Trends Study (NHATS). We focus on methods and assumptions related to attrition and missing data and present a range of scenario analyses that can strengthen conclusions from longitudinal studies of aging.

### Ethics statement

We conducted a secondary analysis of publicly available data (NHATS). Because this is a secondary analysis of data that is in the public domain, informed consent was not obtained for the current study.

### Data source and study population

NHATS is sponsored by the National Institute on Aging (grant number NIA U01AG032947) through a cooperative agreement with the Johns Hopkins Bloomberg School of Public Health [10, 11]. NHATS conducts annual in-home interviews for a diverse, nationally-representative sample of Medicare beneficiaries aged 65 years and older. Our study used longitudinal data from Rounds 1 (2011), 2 (2012), 3 (2013), and 6 (2016) among the initial NHATS cohort that was enrolled in 2011. We restricted our sample to individuals dwelling in the community or non-nursing home residential care settings (e.g., assisted living) at the time of the Round 1 NHATS interview and who participated in primary data collection (i.e., the Sample Person interview) [12].

### Participant characteristics

Baseline characteristics were assessed using Round 1 NHATS survey items. Demographic variables included age, self-reported racial and ethnic category, gender, and residential setting. History of medical conditions, fractures, hospital admissions, surgeries, falls, and use of mobility devices were also described.

### Frailty measures

Frailty was assessed using the Fried frailty phenotype, which defines frailty as a clinical syndrome based on the presence of five clinical signs and symptoms: exhaustion, low physical activity, weakness, slowness, and shrinking [7]. We used the same definitions for these five frailty phenotype components as previously reported in Bandeen-Roche et al. (2015) and best practices outlined in NHATS Technical Documentation [6, 13]. Additional details on measurement of the five frailty phenotype components are provided in S1 Table in S1 File. Individuals were categorized into frailty phenotype states based on the number of frailty components present (robust: 0, prefrail: 1–2, and frail: 3–5) [6, 7]. Frailty state transitions were defined as movements between phenotype categories between interview rounds. Death was considered its own state [14, 15].

### Missing frailty phenotype data

The frailty phenotype is a composite measure, and it was common for individuals to have missing data on one or more of its components. Appropriate approaches for handling missing data require correctly specifying the missing data mechanism. In our case example, we thought data may be missing at random (MAR), where missing values depend on the values of other measured variables. When data are MAR, epidemiologic methods like inverse probability weights or imputation can be used to account for missing data [16–18].

In our analysis, we used two imputation methods to account for missing frailty phenotype data: hot deck imputation and multiple imputation with chained equations. Both of these procedures rely on the assumption that data are MAR and can handle cases when data are not monotonically missing.

Hot deck imputation is a non-parametric missing data approach that imputes missing data using observed values from the underlying data [18]. Values are drawn from the underlying data based on a set of matching variables or covariates. Hot deck imputation relies on the assumptions of (1) exchangeability: individuals with missing data within the stratum of matching variables have the same expected value as units with complete data and (2) positivity: there is at least one observation with complete data in each stratum of matching covariates. In our analysis, individuals with missing information on one or more frailty phenotype component were assigned the frailty phenotype of a randomly matched individual who shared the same pattern for non-missing frailty components but who had no missing frailty data. Hot deck imputation was conducted separately for each round of follow-up in NHATS.

We also conducted analyses using multiple imputation with chained equations (also called multiple imputation with fully conditional specification) to address missing frailty phenotype information [19]. This approach accounts for missing data by fitting a series of iterative prediction models for each of the frailty phenotype components with missing data. Our missing data prediction models included the five frailty phenotype components, as well as residential setting, gender, age, racial and ethnic category, and use of mobility devices. The prediction models are used to fill in the missing values in an iterative process, with the imputed values being updated in each “burn-in” iteration. The full procedure is repeated *m* times to create *m* multiple imputed datasets and results are pooled across the datasets. In our analysis, we created 10 multiple imputed datasets using 10 burn-in iterations.

Multiple imputation with chained equations relies on the assumptions of (1) exchangeability: individuals with missing data within a stratum of measured covariates have the same expected value as units with complete data; (2) positivity: there is at least one observation with complete data in each stratum of measured covariates; and (3) correct specification of the missing data model.

It is possible that in our study, missing frailty phenotype components were at least partially dependent on unobserved component values (i.e., MNAR), in which case hot deck imputation or multiple imputation with chained equations may not fully account for potential bias. As a result, we conducted a series of scenario analyses to describe how various assumptions under an MNAR framework may affect our study results. These analyses are described in detail in the *Scenario Analyses for Missing Data Assumptions* section below.

### Primary analyses of frailty state transitions

One-, two-, and five-year frailty state transitions were visualized using frequency distributions and Sankey Diagrams [20]. Separate Sankey Diagrams were created using hot deck imputation and multiple imputation with chained equations, respectively. In our primary analysis, we aimed to describe the frailty state transitions that would have been observed in the entire population had no one dropped out of the study. We used inverse probability of censoring weights to account for potentially differential loss-to-follow-up [21], upweighting individuals who remained under observation to stand in for similar individuals who were lost-to-follow-up. Because loss-to-follow-up in studies of aging is probably never completely random, approaches to account for informative dropout are preferable to excluding individuals who are lost-to-follow-up.

In our study, the models for the inverse probability of censoring weights included explanatory terms for residential setting, gender, age, racial and ethnic categories, historical medical conditions, healthcare utilization, falls, and mobility devices. The dependent variable was loss-to-follow-up at each timepoint and models were fit separately by baseline frailty phenotype (robust, prefrail, and frail). To fit these models, we excluded a small number of participants with missing covariate data (n = 204, 3%). Importantly, inverse probability of censoring weighting relies on assumptions of (1) exchangeability: units who are censored have the same expected value as units who remain uncensored within strata of measured covariates; (2) positivity: there is at least one observation that remains uncensored within each stratum of measured covariates; and (3) correct specification of the censoring model.

In a separate analysis, loss-to-follow-up was considered a distinct state. Reasons for loss-to-follow-up were described, stratifying by baseline frailty phenotype.

### Scenario analyses for missing data assumptions

We conducted five scenario analyses to assess assumptions regarding missing data and loss-to-follow-up (Table 1). The first three scenario analyses were undertaken to demonstrate how inappropriately addressing missing data and loss-to-follow-up can affect study results. The fourth and fifth scenario analyses calculated plausible values under different assumptions if follow-up data on the frailty phenotype were MNAR. In each scenario, we estimated the proportions of individuals who experienced an improvement, stable, or worsening frailty and the proportion of individuals who died.

**Table 1:** Description of scenario analyses to test assumptions related to missing data and loss-to-follow-up.

Scenario	Individuals missing one or more frailty component at any time ^a^	Individuals lost-to-follow-up	Individuals who died during follow-up
SA 1: Complete case analysis	Excluded	Excluded	Included
SA 2: Exclude individuals who are lost-to-follow-up	Included; hot deck imputation used to account for missing components	Excluded	Included
SA 3: Exclude individuals who died	Included; hot deck imputation used to account for missing components	Excluded	Excluded
SA 4: Last observation carried forward	Included; hot deck imputation used to account for missing components	Included; last observation observed assigned to all unobserved follow-up periods	Included
SA 5: All lost transitioned to frail	Included; hot deck imputation used to account for missing components	Included; participants assigned to frail state for all unobserved follow-up periods	Included

Abbreviations: SA = scenario analysis

^a^ Scenario analyses were based on hot deck imputation and were not conducted for analysis using multiple imputation with chained equations.

In Scenario Analysis 1, we restricted our sample to individuals who had complete information on all frailty components at baseline and during follow-up (complete case analysis), including individuals who died during follow-up. Individuals with missing frailty components and individuals lost-to-follow-up were excluded from all timepoints. In Scenario Analysis 2, we excluded individuals who were lost-to-follow-up from all analyses. Unlike the complete case analysis, missing data on frailty phenotype components were imputed using hot deck imputation. No methods were undertaken to account for differential loss-to-follow-up. In Scenario Analysis 3, we additionally excluded individuals who died from all analyses. These three scenario analyses were undertaken to demonstrate how inappropriately handling missing data can affect study results and we do not recommend these approaches to researchers.

In the Scenario Analysis 4, we used a last-observation-carried-forward approach to impute missing frailty information for those who were lost-to-follow-up; these individuals were assigned their last measured frailty phenotype during all subsequent rounds of follow-up. This analysis represented a scenario in which adults who were lost-to-follow-up never experience frailty progression during the study period. Finally, in Scenario Analysis 5, we conducted an analysis where we assumed that individuals who were lost-to-follow-up transitioned to the frail state, regardless of baseline frailty phenotype. They remained in the frail state for all subsequent rounds. Although taken separately the assumptions in Scenarios 4 and 5 may not be appropriate, taken together in conjunction with our primary analysis, these varying assumptions are useful for describing a range of plausible results in cases where missing data may be MNAR.

## Results

### Study sample

We included 7,608 older adults. Baseline characteristics of the study population are shown in Table 2. After accounting for the NHATS survey sampling weights, 56.6% of individuals were female. Over half of participants were 65–74 years, (52.9%), 33.8% were 75–84 years, and 13.4% were 85+ years. The majority (81.4%) of participants self-identified as non-Hispanic White, 8.2% as non-Hispanic Black, 6.8% as Hispanic, and 3.6% as another racial and ethnic category. The most common medical conditions were history of hypertension (63.9%), arthritis (53.8%), cancer (25.8%), and diabetes (23.8%). Falls (20.0%) and hospital stays (21.0%) during the 12 months prior to the baseline interview were common and 24.1% of individuals used a mobility device.

**Table 2:** Characteristics of community or non-nursing home residential care dwelling older adults at the time of the Round 1 National Health and Aging Trends Study interview ^a^.

Characteristic	n (%)	Accounting for sampling weights (%)
**Demographics**		
Residence		
Community	7,197 (94.6)	(94.5)
Residential care (non-nursing home)	411 (5.4)	(5.5)
Gender		
Male	3,170 (41.7)	(43.4)
Female	4,438 (58.3)	(56.6)
Age category		
65–69	1,408 (18.5)	(27.9)
70–74	1,579 (20.8)	(25.0)
75–79	1,513 (19.9)	(19.1)
80–84	1,505 (19.8)	(14.7)
85–89	953 (12.5)	(9.1)
90+	650 (8.5)	(4.3)
Racial and ethnic categories		
White, non-Hispanic	5,185 (68.9)	(81.4)
Black, non-Hispanic	1,662 (22.1)	(8.2)
Hispanic	454 (6.0)	(6.8)
Other ^b^	225 (3.0)	(3.6)
**Medical history**		
Hypertension	5,107 (67.2)	(63.9)
Arthritis	4,248 (55.9)	(53.8)
Cancer	1,953 (25.7)	(25.8)
Diabetes	1,924 (25.3)	(23.8)
Osteoporosis or thinning of bones	1,559 (20.6)	(21.2)
Heart disease (including angina or CHF)	1,411 (18.6)	(17.5)
Myocardial infarction	1,164 (15.3)	(14.1)
Lung disease	1,154 (15.2)	(15.4)
Stroke	892 (11.7)	(10.0)
Dementia or Alzheimer’s Disease	457 (6.0)	(4.4)
**History of fractures or falls**		
Hip fracture (since age 50)	379 (5.0)	(4.1)
Other fracture (since age 50)	1,519 (20.0)	(20.2)
Fallen in last month	832 (10.9)	(10.4)
Worry about falling in last month	2,251 (29.6)	(27.4)
Fallen in last 12 months	1,547 (20.4)	(20.0)
**Healthcare utilization and surgeries in last 12 months**		
Hospital stay	1,777 (23.4)	(21.0)
Cataract surgery	463 (6.1)	(5.8)
Heart surgery	155 (2.0)	(2.1)
Knee surgery	99 (1.3)	(1.5)
Hip surgery	68 (0.9)	(0.8)
Back or spine surgery	54 (0.7)	(0.8)
**Mobility or walking devices in last month**		
Any mobility device or walking device	2,322 (30.5)	(24.1)
Cane	1,602 (21.1)	(16.4)
Walker	1,146 (15.1)	(11.6)
Wheelchair	597 (7.9)	(6.1)
Scooter	196 (2.6)	(2.3)

Abbreviations: CHF = congestive heart failure.

^a^ Number of individuals with missing data for each covariate: racial and ethnic category (82), hypertension (10), arthritis (16), cancer (3), diabetes (3), osteoporosis or thinning of bones (27), heart disease (16), myocardial infarction (8), lung disease (5), stroke (8), dementia/Alzheimer’s Disease (6), hip fracture (4), other fracture (6), worry about falling in last month (12), fallen in last 12 months (12), hospital stay (9), cataract surgery (11), heart surgery (10), knee surgery (2), hip surgery (2), back or spine surgery (5), and mobility devices (3).

^b^ Other category includes American Indian, Asian, Native Hawaiian, Pacific Islander, other non-Hispanic racial category, and individuals who reported more than one racial and ethnic category without specifying primary.

### Frailty phenotype

One or more frailty phenotype components were missing at baseline for 14.8% of the study participants (S2 Table in S1 File). The components with the most missing data across all time periods were physical objective measures, including weakness (range: 7.8–11.3%) and slowness (range: 5.8–9.8%), followed by shrinking (range: 3.3–4.3%). Less than 1% of individuals were missing data on self-reported exhaustion or low physical activity across all time periods. Baseline characteristics of the study population for individuals with and without missing frailty phenotype components are presented in S3 Table in S1 File. A higher proportion of older adults with missing frailty phenotype information were Black, resided in residential care settings, and reported using mobility devices than among those without missing frailty phenotype information. After accounting for the NHATS survey sampling weights and hot deck imputation, 39.7% of individuals were classified as robust, 45.6% as prefrail, and 14.8% as frail at baseline. The proportions were similar when using multiple imputation with chained equations (robust: 39.2%, prefrail: 45.5%, frail 15.4%).

### Loss-to-follow-up

Loss-to-follow-up was 15.8% at 1-year post baseline, 26.1% at 2-years, and 37.4% at 5-years. Baseline characteristics of the study sample by response status is provided in S4 Table in S1 File. The baseline characteristics among study participants who were lost-to-follow-up at 1-, 2-, and 5-years post-baseline were similar to those who participated in the follow-up interviews. A higher proportion of individuals who were lost to follow-up 5-years post-baseline were frail at baseline (12.7%) compared to those who remained in the study (9.0%).

Proportions of loss-to-follow-up at 1- and 2-years post-baseline were similar by baseline frailty phenotype; however the reasons for loss-to-follow-up differed (Table 3). Across time periods, frail individuals were more likely to be lost-to-follow-up due to a physical or mental inability to attend the study visit than their robust or prefrail counterparts. Alternatively, robust individuals had the highest proportion of loss-to-follow-up due to refusal to participate. One-, two-, and five-year frailty state transitions incorporating loss-to-follow-up as a state are provided in S1 Fig in S1 File.

**Table 3:** Reasons for loss-to-follow-up, stratified by baseline frailty phenotype ^a^.

Reason for loss-to-follow-up	Baseline frailty		
	Robust	Prefrail	Frail
**1-year post-baseline, n (%)**	**413 (15.9)**	**571 (15.8)**	**191 (13.6)**
Facility questionnaire only ^b^	11 (2.7)	26 (4.6)	20 (10.5)
Physically/mentally unable or too ill to participate	14 (3.4)	39 (6.8)	20 (10.5)
Refusal	361 (87.4)	461 (80.7)	140 (73.3)
Ineligible ^c^	7 (1.7)	23 (4.0)	4 (2.1)
Other ^d^	20 (4.8)	22 (3.9)	7 (3.7)
**2-years post-baseline, n (%)**	**695 (26.8)**	**931 (25.8)**	**297 (21.2)**
Facility questionnaire only ^b^	13 (1.9)	41 (4.4)	22 (7.4)
Physically/mentally unable or too ill to participate	25 (3.6)	76 (8.2)	39 (13.1)
Refusal	601 (86.5)	735 (78.9)	215 (72.4)
Ineligible ^c^	13 (1.9)	35 (3.8)	5 (1.7)
Other ^d^	43 (6.2)	44 (4.7)	16 (5.4)
**5-years post-baseline, n (%)**	**1022 (39.4)**	**1325 (36.7)**	**404 (28.9)**
Facility questionnaire only ^b^	13 (1.3)	38 (2.9)	21 (5.2)
Physically/mentally unable or too ill to participate	55 (5.4)	129 (9.7)	66 (16.3)
Refusal	864 (84.5)	1035 (78.1)	281 (69.6)
Ineligible ^c^	20 (2.0)	49 (3.7)	8 (2.0)
Other ^d^	70 (6.8)	74 (5.6)	28 (6.9)

Notes

^a^ Percentages do not account for NHATS survey sampling weights.

^b^ The participating individuals were included in the NHATS round through a Facility Questionnaire only that was filled out by a staff member of the respective residential-care setting. The Individual did not participate in the primary data collection instrument, the Sample Person Questionnaire.

^c^ Ineligible category includes people who moved out of Primary Sampling Unit or out of the contiguous US.

^d^ Other category includes participants with a language barrier, those who were unavailable or unable to be located, and those with other reasons.

### Frailty state transitions using inverse probability weighting to account for loss-to-follow-up

**Fig 1:**
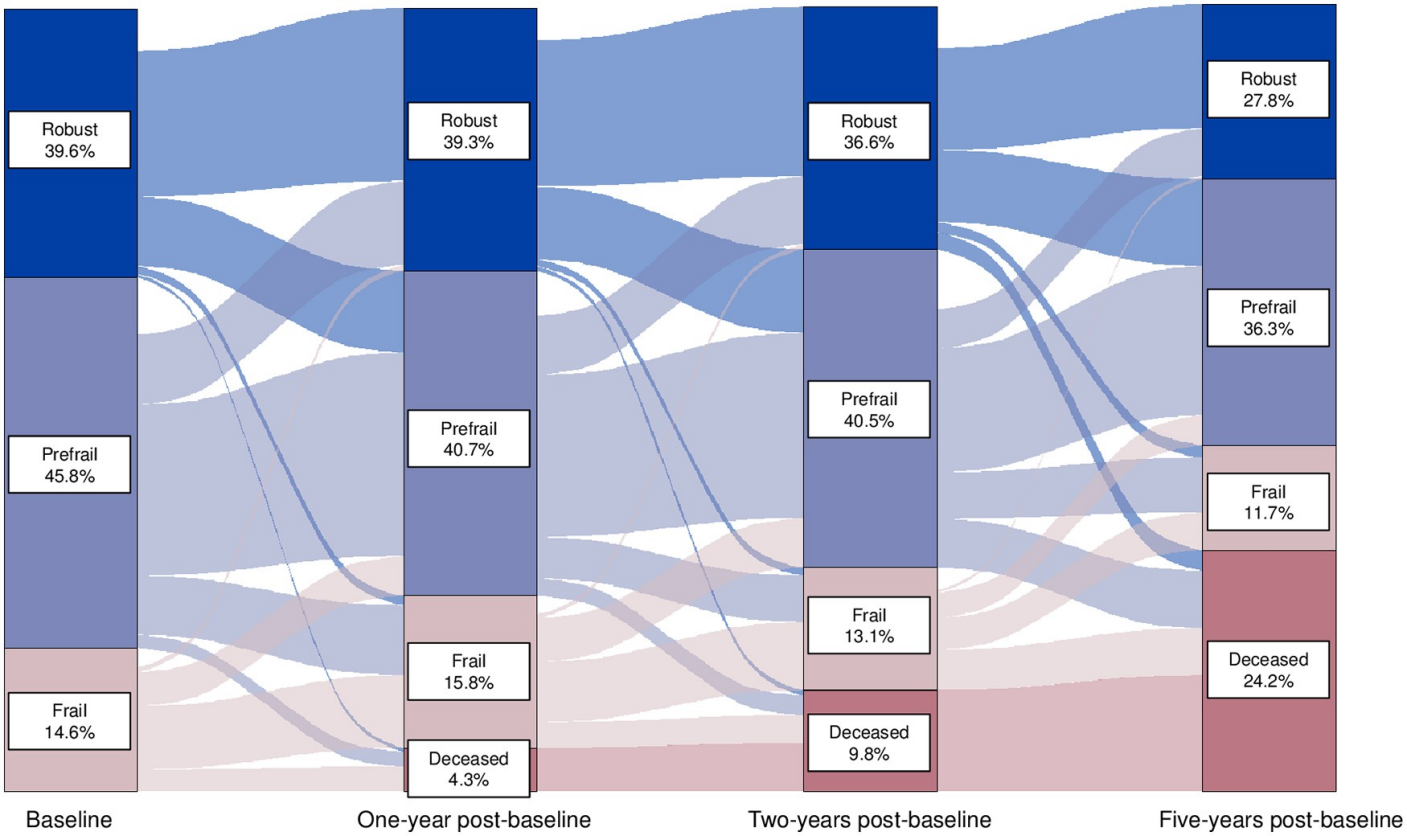
Sankey diagram of frailty state transitions using inverse probability of censoring weighting to address potential informative censoring.

A Sankey diagram presenting 1-, 2-, and 5-year frailty state transitions, after hot deck imputation and accounting for potentially informative censoring using inverse probability weighting, is presented in Fig 1. The distribution of censoring weights by year of follow-up are presented in S2 Fig in S1 File. At one-year, most individuals remained in the same frailty phenotype category: robust (68.1%), prefrail (55.3%), and frail (49.5%). Transitions between frailty states became more common with longer follow-up. Across all time periods, transitions between adjacent frailty phenotype categories (i.e., robust to/from prefrail, prefrail to/from frail) were more common than transitions across multiple phenotype categories (i.e., robust to frail, frail to robust). At five years, 49.8% of the robust participants at baseline remained robust, 34.2% were prefrail, 5.3% were frail, and 10.7% were deceased. For prefrail individuals at baseline, 17.0% had improved to the robust state after five years, 13.2% had worsened to the frail state, and 25.8% were deceased. For frail individuals at baseline, over half (55.3%) of frail individuals at baseline were deceased by five years, 2.3% had transitioned to the robust state, and 18.1% had transitioned to the prefrail state. The results were similar when using multiple imputation with chained equations to account for missing frailty phenotype information (S3 Fig in S1 File).

### Scenario analysis results

Results from the scenario analyses to test assumptions regarding loss-to-follow-up are presented in Table 4. Less than half of participants had all frailty phenotype components measured across all timepoints (n = 3,249). More individuals were categorized as robust (43.2%) at baseline when using a complete case approach (Scenario Analysis 1), compared to hot deck imputation, and fewer were identified as frail (12.1%). The proportions of individuals who experienced improvement, stable, or worsening frailty or death were similar when using inverse probability weighting and the complete case analysis. The distributions were also similar when we excluded all individuals who were lost-to-follow-up (Scenario Analysis 2). When we excluded individuals who died, a higher proportion of individuals were classified as experiencing improvement or stable frailty state across all timepoints, with larger discrepancies occurring over longer duration of follow-up (Scenario Analysis 3).

**Table 4:** Results of scenario analyses to test assumptions related to missing data and loss-to-follow-up.

Scenario analysis	Improvement	Stable	Worsening	Deceased
**1-year post-baseline, n (%)**				
Primary: Inverse probability weighting	16.6	59.5	19.5	4.3
SA1: Complete case analysis				
SA2: Exclude individuals who are lost-to-follow-up	16.2	58.3	19.3	6.2
SA3: Exclude individuals who died	17.3	62.1	20.6	-
SA4: Last-observation-carried-forward	13.7	65.6	16.9	3.8
SA5: All-lost-transitioned-to-frail	13.7	52.0	30.5	3.8
**2-years post-baseline, n (%)**				
Primary: Inverse probability weighting	16.3	53.5	20.4	9.8
SA1: Complete case analysis				
SA2: Exclude individuals who are lost-to-follow-up	15.6	51.8	19.9	12.7
SA3: Exclude individuals who died	17.9	59.4	22.8	-
SA4: Last-observation-carried-forward	13.5	60.9	17.7	8.0
SA5: All-lost-transitioned-to-frail	11.7	42.4	37.9	8.0
**5-years post-baseline, n (%)**				
Primary: Inverse probability weighting	10.7	43.4	21.7	24.2
SA1: Complete case analysis				
SA2: Exclude individuals who are lost-to-follow-up	10.4	42.0	21.5	26.1
SA3: Exclude individuals who died	14.1	56.8	29.1	-
SA4: Last-observation-carried-forward	10.2	54.5	18.6	16.7
SA5: All-lost-transitioned-to-frail	6.5	30.8	46.0	16.7

Abbreviations: SA = scenario analysis.

The last-observation-carried-forward approach led to lower estimates of mortality or worsening frailty at all time points, and higher classification of stable frailty (Scenario Analysis 4). As expected, when we classified all individuals who were lost-to-follow-up as transitioning to the frail state, we estimated substantially more worsening frailty and less improvement or stable frailty than when using inverse probability weighting at all time points (Scenario Analysis 5).

## Discussion

We describe important methodological considerations and potential solutions when accounting for missing data and loss-to-follow-up in longitudinal studies of aging. We demonstrate how researchers might use these analytical tools by presenting a case example describing five-year frailty state transitions in a contemporary cohort representative of US Medicare beneficiaries 65 years of age or older.

In the NHATS cohort, we found that frailty state transitions were common. Although transitions to worsening frailty states occurred more often, especially over longer periods of follow-up, improvements were also common. Patterns of frailty state transitions varied substantially based on the baseline frailty phenotype, with prefrail and frail individuals experiencing quicker progression to worse frailty states or death than robust individuals. Prefrail individuals were more likely than frail individuals to experience frailty improvements over time [22]. This highlights the need for early detection of prefrailty, through use of comprehensive geriatric assessment or other frailty screening tools [23], to help target interventions when they may be most effective.

Although other studies have described frailty state transitions in older community-dwelling adult populations, many of these studies excluded individuals who died, were loss-to-follow-up, or were missing data on frailty from their study denominators [22, 24–28]. It is critical to use appropriate epidemiologic methods to account for the potential biases that these exclusions introduce. As a first step, describing patterns of missing data can help researchers during the study design phase and when interpreting findings. In our case example, we found that while missing data on frailty phenotype components that are captured exclusively via self-reported measures (exhaustion and low physical activity) were rare, missing data on components based on physical assessments were common across all study timepoints. This is not surprising, given that many older adults may face barriers to participating in performance-based assessments.

It is also important to carefully consider the underlying missing data mechanism. A missing completely at random (MCAR) missing data mechanism, in which missing data are not related to observed or unmeasured variables, is unlikely in studies of aging. This assumption is also easy to refute in our case example since we observed that baseline characteristics differed among those with and without missing frailty phenotype components [2]. Complete case analyses may result in bias when data are not MCAR. In our primary analysis, we used hot deck imputation to probabilistically impute missing values for the frailty phenotype components. Hot deck imputation resulted in higher proportions of frail individuals and lower proportions of robust individuals at baseline compared to a complete case analysis. An analysis using multiple imputation with chained equations produced similar results. Researchers should use appropriate methods like hot deck imputation [18], multiple imputation with chained equations [19], or inverse probability of missingness weighting [29], which can help mitigate potential bias when missingness mechanisms can be accounted for using measured covariates (i.e., MAR). However, when the missing data mechanism depends on unobserved variables (i.e., MNAR), these approaches may not fully account for potential bias. In these cases, scenario analyses that represent “best case” and “worst case” scenarios can shed light on whether interpreted findings hold even in cases of differential missing data.

In addition to missing data on the frailty phenotype, we also found that loss-to-follow-up was substantial in the NHATS cohort. Although the proportions lost-to-follow-up were similar across frailty categories at one- and two-years post-baseline according to baseline frailty phenotype, the reasons for loss-to-follow-up differed. Frail individuals were more likely to be lost-to-follow-up due to physical or mental inabilities than their robust or prefrail counterparts and robust individuals were more likely to refuse to participate than prefrail or frail individuals. NHATS does not collect information on the reason for refusal. The proportions lost-to-follow-up diverged greatly across baseline frailty phenotypes for longer follow-up durations, with robust individuals being more likely to be lost-to-follow-up than prefrail or frail participants. Alternatively, individuals who were prefrail or frail were more likely to die. It is critical in longitudinal studies of older adults to consider the reasons and implications for loss-to-follow-up and mortality, including the potential for bias.

We opted to exclude individuals with missing covariate data when calculating the inverse probability of censoring weights and in the multiple imputation with chained equations models, since the proportion with any missing covariate data was small (3%) and unlikely to impact results. Missingness for each of the individual covariates was ≤1%. The amount of bias or precision loss that results from conducting a complete case analysis depends on the extent of missing data. Researchers should weigh the relative tradeoffs between simplicity, computational efficiency, and risk of bias when considering how to handle missingness for variables with a small amount of missingness [30].

We tested several assumptions regarding loss-to-follow-up in a series of scenario analyses. Our main results using inverse probability of censoring weighting were similar to results that excluded individuals who were lost-to-follow-up. This may be due to misspecification of the censoring weight model or due to unmeasured predictors of loss-to-follow-up. Linkage between the NHATS cohort and Medicare insurance claims and enrollment data may allow further refinement of models to account for informative loss-to-follow-up in future work. Alternatively, loss-to-follow-up in the NHATS cohort may truly be non-informative, which could explain the similarity between results.

We also found that excluding individuals who died led to higher proportions of individuals being classified as experiencing “improvement” in frailty over time. Prior studies that excluded individuals who died during follow-up tended to report more favorable frailty trajectories than those that explicitly considered death in their analyses [24–26]. When describing and modeling health trajectories in older adults, it is critical to account for death, which is an undeniable aspect of the aging process [14, 15]. In some cases, it may be more appropriate to treat death as a competing event [31, 32], rather than as a state or outcome in a model. We strongly urge researchers never to exclude individuals who die during follow-up from analyses in studies of older adults, since this is likely to result in bias.

Our final two scenario analyses (last-observation-carried-forward, all-lost-transitioned-to-frail) also led to substantially different results than our analyses using inverse probability weighting. In conjunction, these approaches are similar to a “bounds” analysis, where researchers may set a range of plausible values around estimates. In studies of intervention effects, bounds are typically encoded differentially with respect to an exposure [33, 34]. We recommend that researchers conducting longitudinal analyses in older adult populations test a range of assumptions related to censoring and loss-to-follow-up.

## Conclusions

Our study presents methodological challenges related to missing data in studies of aging using a case example describing five-year frailty state transitions in a diverse cohort of older Medicare beneficiaries in the United States. Our results highlight the importance of rigorous epidemiologic methodology in studies of aging, as the implications of missing data, death, and loss-to-follow-up can be substantial in these populations. We urge researchers to be transparent about the quality of data and extent of missingness in their studies, and to use epidemiologic tools to mitigate potential bias.

## Supporting information

S1 File File of supporting tables and figures. (DOCX)
