# Supplementary material for: Missing data approaches in longitudinal studies of aging: A case example using the National Health and Aging Trends Study
Source: PLoS One. 2023 Jun 8;18(6):e0286984. doi: 10.1371/journal.pone.0286984 (PMC10249888; doi:10.1371/journal.pone.0286984)

**Missing data approaches in longitudinal studies of aging: A case example using the National Health and Aging Trends Study**

**Supplemental material**

**S1 Table. Definitions for frailty phenotype components in the NHATS data**

| **Frailty phenotype component** | **NHATS definition** |
| --- | --- |
| Exhaustion | “Yes” to question “In the last month, did you have low energy or were you easily exhausted?” (variable: ss#lowenergy), AND  “Yes” to question “In the last month, did your low energy or exhaustion ever limit your activities?” (variable: ss#loenlmtat) |
| Low physical activity | “No” to question “In the last month did you ever go walking for exercise?” (variable: pa#evrgowalk), AND  “No” to question “In the last month, did you ever spend time on vigorous activities that increased your heart rate and made you breath harder? This includes things like working out, swimming, running, or biking, or playing a sport.” (variable: pa#vigoractv) |
| Shrinking | BMI < 18.5kg/m^2^ BMI was calculated using the self-reported height (hw#howtallft and hw#howtallin) and weight (hw#currweigh) and the standard conversion formula  $BMI=\frac{weight (lb) \times703}{\left[ height \left( in \right) \right]^{2}}$ OR  “Yes” to question “Have you lost 10 or more pounds in the last 12 months?” (variable: hw#lst10pnds) AND “No” to question “Were you trying to lose weight?” (variable: hw#trytolose) |
| Slowness | First walking speed trial: lowest 20% (by sex and height)  Walking speed in meters per second was assessed using the SAS code provided in the NHATS Technical Paper #4. Individuals who attempted but did not complete the walking test or who were ineligible due to safety received a score of 0 meters per second. |
| Weakness | Maximum dominant hand grip strength trial: lowest 20% (by sex and BMI)  Grip strength was assessed using the SAS code provided in the NHATS Technical Paper #4. Individuals who attempted but did not complete the walking test or who were ineligible due to safety received a score of 0. |

Abbreviations: BMI=body mass index; NHATS=National Health and Aging Trends Study.

References:

Bandeen-Roche K, Seplaki CL, Huang J, et al. Frailty in Older Adults: A Nationally Representative Profile in the United States. *J Gerontol A Biol Sci Med Sci* 2015; 70: 1427-1434. 2015/08/25. DOI: 10.1093/gerona/glv133.Fried LP, Tangen CM, Walston J, et al. Frailty in older adults: evidence for a phenotype. *J Gerontol A Biol Sci Med Sci* 2001; 56: M146-156. 2001/03/17. DOI: 10.1093/gerona/56.3.m146.

Niefeld MR. SAS programming statements for construction of performancebased summary measures of physical capacity in the National Health and Aging Trends Study. Addendum to NHATS Technical Paper #4. Baltimore: Johns Hopkins University School of Public Health2012.

S2 Table. Summary of missing data on the frailty phenotype components

| **Component** | **Baseline** | **One-year post-baseline** | **Two-years post-baseline** | **Five-years post-baseline** |
| --- | --- | --- | --- | --- |
| **Number of missing frailty components** | | | | |
| 0 | 6,483 (85.2) | 5,110 (84.4) | 4,162 (85.2) | 2,839 (88.4) |
| 1 | 680 (8.9) | 536 (8.9) | 355 (7.3) | 210 (6.5) |
| 2 | 276 (3.6) | 297 (4.9) | 270 (5.5) | 110 (3.4) |
| 3 | 164 (2.2) | 108 (1.8) | 91 (1.9) | 54 (1.7) |
| 4 | 5 (0.1) | 4 (0.1) | 5 (0.1) | 0 (0.0) |
| **Exhaustion** |  |  |  |  |
| Yes | 2,371 (31.2) | 1,878 (31.0) | 1,503 (30.8) | 1,013 (31.5) |
| No | 5,225 (68.7) | 4,157 (68.6) | 3,357 (68.7) | 2,187 (68.1) |
| Missing | 13 (0.2) | 21 (0.3) | 24 (0.5) | 13 (0.4) |
| **Low physical activity** |  |  |  |  |
| Yes | 2,621 (34.4) | 2,083 (34.4) | 1,619 (33.1) | 1,115 (34.7) |
| No | 4,982 (65.5) | 3,967 (65.5) | 3,254 (66.6) | 2,095 (65.2) |
| Missing | 6 (0.1) | 6 (0.1) | 11 (0.2) | 3 (0.1) |
| **Slowness** |  |  |  |  |
| Yes | 1,821 (23.9) | 1,517 (25.0) | 1,189 (24.3) | 817 (25.4) |
| No | 5,046 (66.3) | 3,996 (66.0) | 3,288 (67.3) | 2,209 (68.8) |
| Missing | 742 (9.8) | 543 (9.0) | 407 (8.3) | 187 (5.8) |
| **Shrinking** |  |  |  |  |
| Yes | 1,313 (17.3) | 1,079 (17.8) | 937 (19.2) | 613 (19.1) |
| No | 6,042 (79.4) | 4,746 (78.4) | 3,748 (76.7) | 2,461 (76.6) |
| Missing | 254 (3.3) | 231 (3.8) | 199 (4.1) | 139 (4.3) |
| **Weakness** |  |  |  |  |
| Yes | 1,607 (21.1) | 1,183 (19.5) | 979 (20.0) | 715 (22.3) |
| No | 5,268 (69.2) | 4,202 (69.4) | 3,353 (68.7) | 2,248 (70.0) |
| Missing | 734 (9.6) | 671 (11.1) | 552 (11.3) | 250 (7.8) |

Note: Percentages are taken among people who are not deceased or not lost to follow-up at the time of the measurement. Percentages do not account for NHATS survey sampling weights.

**S3 Table. Characteristics of community or on-nursing home residential care dwelling older adults at the time of the Round 1 National Health and Aging Trends Study interview, by missingness in the frailty phenotype components at baseline ^a^**

| **Characteristic** | **Missing ≥1 frailty phenotype components** | **No missing frailty phenotype components** |
| --- | --- | --- |
| **Demographics** | | |
| Residence |  |  |
| Community | 88.6 | 95.4 |
| Residential care (non-nursing home) | 11.4 | 4.6 |
| Gender |  |  |
| Male | 38.1 | 44.2 |
| Female | 61.9 | 55.8 |
| Age category |  |  |
| 65-69 | 21.3 | 28.9 |
| 70-74 | 21.8 | 25.4 |
| 75-79 | 17.8 | 19.3 |
| 80-84 | 16.6 | 14.4 |
| 85-89 | 14.6 | 8.3 |
| 90+ | 7.9 | 3.7 |
| Racial and ethnic categories |  |  |
| White, non-Hispanic | 72.9 | 82.6 |
| Black, non-Hispanic | 11.3 | 7.8 |
| Hispanic | 9.1 | 6.5 |
| Other ^b^ | 6.6 | 3.1 |
| **Medical history** | | |
| Hypertension | 64.7 | 63.8 |
| Arthritis | 55.7 | 53.5 |
| Cancer | 21.3 | 26.5 |
| Diabetes | 26.4 | 23.5 |
| Osteoporosis or thinning of bones | 22.5 | 21.0 |
| Heart disease (including angina or CHF) | 17.6 | 17.4 |
| Myocardial infarction | 16.0 | 13.8 |
| Lung disease | 12.8 | 15.8 |
| Stroke | 12.9 | 9.6 |
| Dementia or Alzheimer's Disease | 12.0 | 3.3 |
| **History of fractures or falls** | | |
| Hip fracture (since age 50) | 7.0 | 3.7 |
| Other fracture (since age 50) | 20.1 | 20.2 |
| Fallen in last month | 14.4 | 9.8 |
| Worry about falling in last month | 34.3 | 26.4 |
| Fallen in last 12 months | 20.8 | 19.9 |
| **Healthcare utilization and surgeries in last 12 months** | | |
| Hospital stay | 27.0 | 20.1 |
| Cataract surgery | 6.2 | 5.8 |
| Heart surgery | 2.1 | 2.2 |
| **Mobility or walking devices in last month** | | |
| Any mobility device or walking device | 38.3 | 21.9 |
| Cane | 22.6 | 15.5 |
| Walker | 20.7 | 10.3 |
| Wheelchair | 12.9 | 5.0 |
| Scooter | 3.3 | 2.2 |

CHF=congestive heart failure; LTFU=lost-to-follow-up.
^a^ Percentages account for the NHATS survey sampling design.
^b^ Other category includes American Indian, Asian, Native Hawaiian, Pacific Islander, other non-Hispanic racial category, and individuals who reported more than one racial and ethnic category without specifying primary.

S4 Table. Characteristics of community or on-nursing home residential care dwelling older adults at the time of the Round 1 National Health and Aging Trends Study interview, by participation in follow-up surveys ^a^

| **Characteristic** | **1-year follow-up (%)** | | | **2-year follow-up (%)** | | | **5-year follow-up (%)** | | |
| --- | --- | --- | --- | --- | --- | --- | --- | --- | --- |
|  | **Participated** | **LTFU** | **Deceased** | **Participated** | **LTFU** | **Deceased** | **Participated** | **LTFU** | **Deceased** |
| **Demographics** |  |  |  |  |  |  |  |  |  |
| Residence |  |  |  |  |  |  |  |  |  |
| Community | 94.6 | 96.0 | 85.6 | 95.0 | 96.4 | 84.0 | 96.0 | 97.2 | 84.3 |
| Residential care (non-nursing home) | 5.4 | 4.0 | 14.4 | 5.0 | 3.6 | 16.0 | 4.0 | 2.8 | 15.7 |
| Sex |  |  |  |  |  |  |  |  |  |
| Male | 43.7 | 41.5 | 45.1 | 43.0 | 44.1 | 44.3 | 42.5 | 43.7 | 45.0 |
| Female | 56.3 | 58.5 | 54.9 | 57.0 | 55.9 | 55.7 | 57.5 | 56.3 | 55.0 |
| Age category |  |  |  |  |  |  |  |  |  |
| 65-69 | 28.6 | 28.6 | 10.3 | 29.3 | 30.0 | 9.2 | 33.0 | 29.5 | 10.5 |
| 70-74 | 25.0 | 27.5 | 12.8 | 25.6 | 27.0 | 13.0 | 26.9 | 27.9 | 13.3 |
| 75-79 | 19.2 | 18.9 | 16.9 | 19.2 | 19.9 | 15.8 | 19.3 | 19.8 | 16.8 |
| 80-84 | 14.5 | 13.7 | 23.1 | 14.4 | 13.0 | 22.6 | 12.5 | 13.4 | 23.5 |
| 85-89 | 8.7 | 8.3 | 20.7 | 8.2 | 7.3 | 22.6 | 6.5 | 7.1 | 20.9 |
| 90+ | 3.9 | 2.9 | 16.2 | 3.3 | 2.8 | 16.9 | 1.8 | 2.4 | 15.1 |
| Racial and ethnic categories |  |  |  |  |  |  |  |  |  |
| White, non-Hispanic | 82.0 | 78.4 | 82.1 | 83.2 | 76.5 | 83.1 | 84.3 | 77.3 | 82.5 |
| Black, non-Hispanic | 8.1 | 8.6 | 7.9 | 7.8 | 9.2 | 8.7 | 7.5 | 8.8 | 8.9 |
| Hispanic | 6.5 | 8.0 | 7.6 | 6.0 | 9.1 | 5.9 | 5.5 | 8.9 | 5.8 |
| Other ^b^ | 3.3 | 5.0 | 2.5 | 3.0 | 5.3 | 2.3 | 2.7 | 5.0 | 2.8 |
| **Medical history** |  |  |  |  |  |  |  |  |  |
| Hypertension | 63.8 | 62.9 | 70.7 | 63.7 | 63.2 | 68.4 | 63.0 | 63.3 | 67.8 |
| Arthritis | 53.8 | 52.2 | 61.0 | 53.7 | 51.5 | 62.0 | 53.6 | 51.5 | 59.4 |
| Cancer | 25.7 | 24.1 | 35.6 | 25.3 | 24.3 | 35.6 | 24.2 | 24.6 | 33.1 |
| Diabetes | 23.6 | 22.7 | 32.7 | 23.4 | 23.1 | 29.9 | 22.9 | 22.8 | 28.7 |
| Osteoporosis or thinning of bones | 21.1 | 21.3 | 23.3 | 21.1 | 20.3 | 24.8 | 21.3 | 20.0 | 23.8 |
| Heart disease (including angina or CHF) | 17.1 | 14.6 | 35.7 | 16.5 | 15.1 | 32.8 | 15.2 | 15.0 | 29.2 |
| Myocardial infarction | 13.7 | 13.5 | 25.4 | 13.0 | 12.9 | 26.8 | 11.5 | 12.8 | 23.9 |
| Lung disease | 14.7 | 15.5 | 30.3 | 14.2 | 15.4 | 26.0 | 14.0 | 14.3 | 21.7 |
| Stroke | 9.7 | 10.0 | 16.3 | 9.5 | 9.1 | 17.0 | 8.1 | 9.0 | 17.8 |
| Dementia or Alzheimer's Disease | 3.8 | 4.2 | 18.6 | 3.2 | 3.4 | 17.5 | 2.2 | 2.9 | 13.9 |
| **History of fractures or falls** |  |  |  |  |  |  |  |  |  |
| Hip fracture (since age 50) | 3.9 | 3.5 | 11.2 | 3.6 | 3.6 | 10.0 | 3.1 | 3.2 | 9.0 |
| Other fracture (since age 50) | 20.2 | 19.1 | 23.1 | 20.7 | 18.2 | 22.6 | 20.3 | 19.1 | 22.0 |
| Fallen in last month | 9.8 | 9.7 | 26.4 | 9.4 | 9.0 | 23.3 | 9.3 | 8.7 | 17.2 |
| Worry about falling in last month | 26.6 | 26.3 | 50.3 | 26.3 | 24.3 | 46.9 | 24.6 | 24.1 | 42.7 |
| Fallen in last 12 months | 20.2 | 17.7 | 25.6 | 20.5 | 17.2 | 25.4 | 20.0 | 17.8 | 25.2 |
| **Healthcare utilization and surgeries in last 12 months** | | | | | | | | | |
| Hospital stay | 20.6 | 16.5 | 49.8 | 19.8 | 17.9 | 41.7 | 17.1 | 18.5 | 37.5 |
| Cataract surgery | 6.0 | 5.9 | 2.2 | 6.0 | 5.9 | 4.0 | 5.7 | 5.7 | 6.5 |
| Heart surgery | 2.1 | 2.4 | 2.4 | 2.0 | 2.5 | 2.2 | 1.8 | 2.4 | 2.7 |
| **Mobility or walking devices in last month** | | | | | | | | | |
| Any mobility device or walking device | 22.8 | 21.0 | 63.3 | 21.2 | 20.5 | 59.9 | 17.9 | 19.1 | 52.0 |
| Cane | 16.0 | 15.6 | 28.7 | 15.2 | 15.4 | 29.6 | 13.7 | 14.5 | 28.1 |
| Walker | 10.8 | 8.1 | 43.3 | 9.7 | 8.4 | 38.0 | 7.3 | 8.0 | 31.7 |
| Wheelchair | 5.2 | 4.5 | 30.8 | 4.6 | 4.3 | 23.5 | 3.2 | 3.9 | 18.6 |
| Scooter | 2.2 | 1.5 | 7.0 | 2.1 | 1.7 | 5.8 | 1.7 | 1.9 | 4.8 |
| **Frailty phenotype** | | | | | | | | | |
| Robust | 41.1 | 39.3 | 10.1 | 42.5 | 41.0 | 11.4 | 46.2 | 41.9 | 16.7 |
| Prefrail | 45.8 | 46.9 | 36.1 | 45.8 | 45.7 | 43.3 | 44.8 | 45.4 | 48.1 |
| Frail | 13.1 | 13.8 | 53.8 | 11.7 | 13.3 | 45.3 | 9.0 | 12.7 | 35.2 |

CHF=congestive heart failure; LTFU=lost-to-follow-up.
^a^ Percentages account for the NHATS survey sampling design.
^b^ Other category includes American Indian, Asian, Native Hawaiian, Pacific Islander, other non-Hispanic racial category, and individuals who reported more than one racial and ethnic category without specifying primary.

S1 Fig. Sankey diagram of frailty state transitions with loss-to-follow-up as a category

**
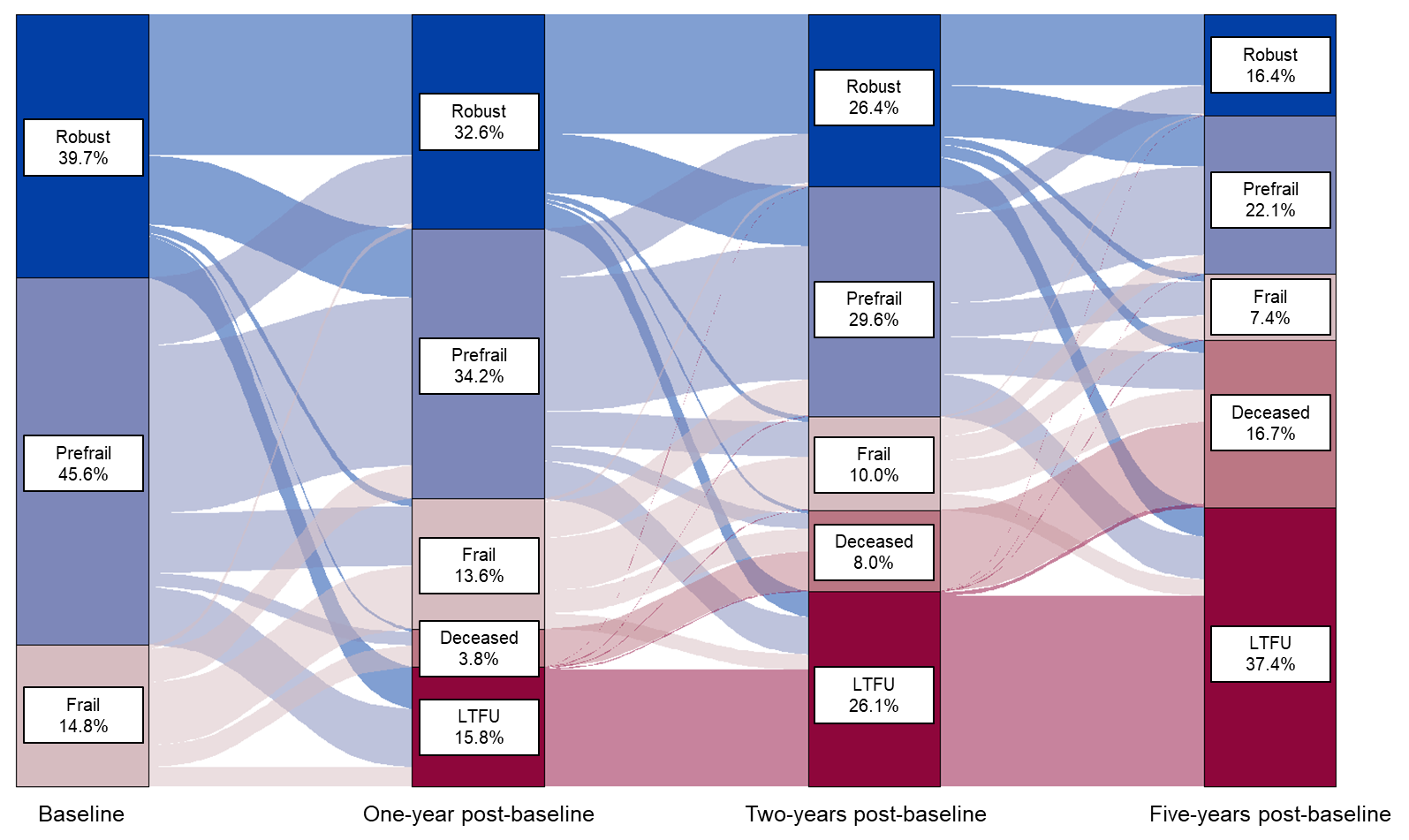
**

Abbreviations: LTFU=loss-to-follow-up

**S2 Fig. Distribution of inverse probability of censoring weights to account for potentially informative loss-to-follow-up**
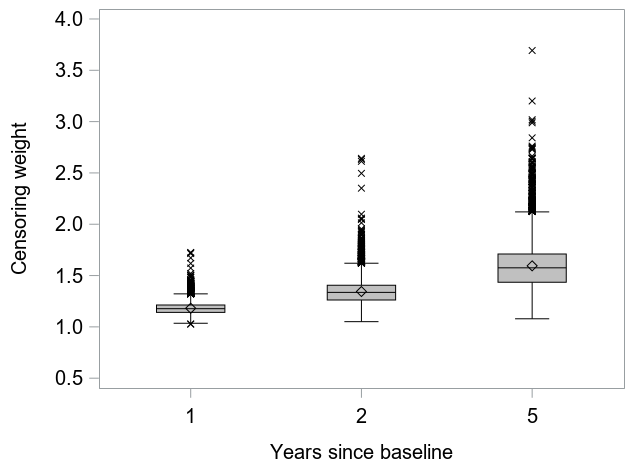


S3 Fig. Sankey diagram of frailty state transitions using multiple imputation with chained equations to account for missing frailty phenotype information and inverse probability of censoring weights to account for loss to follow-up


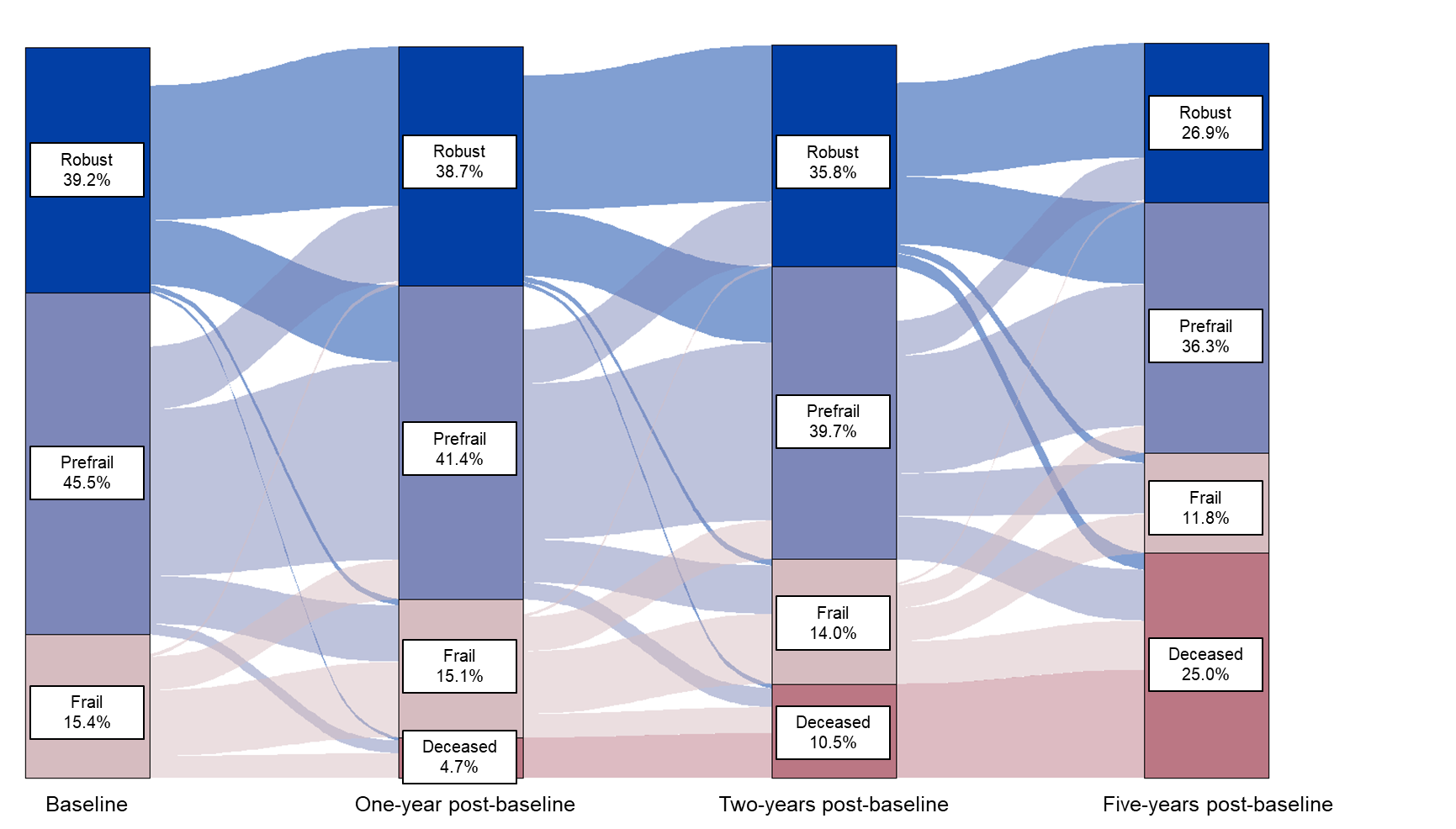

Supplement: S1 File — (DOCX) [file pone.0286984.s001.docx]
